# Teleophthalmology and Remote Eye Care: A Bibliometric Analysis of the 50 Most Highly Cited Articles From 2000 to 2025

**DOI:** 10.7759/cureus.112827

**Published:** 2026-07-16

**Authors:** Norah K Alshahrani, Maryam B Alqudihi, Abdulaziz W Aboajmah, Alanoud R Alsubaie, Marwa A Akram, Tif S Alghamdi, Abdulrahman Alamri

**Affiliations:** 1 Medical School, College of Medicine, King Khalid University, Abha, SAU; 2 Ophthalmology, King Faisal University, Hafuf, SAU; 3 Ophthalmology, College of Medicine, Al-Rayan Colleges, Madinah, SAU; 4 Ophthalmology, Imam Abdulrahman Bin Faisal University, Dhahran, SAU; 5 Faculty of Medicine, Ataturk University, Erzurum, TUR; 6 Ophthalmology, University of Jeddah, Jeddah, SAU; 7 Ophthalmology, College of Medicine, King Khalid University, Abha, SAU

**Keywords:** artificial intelligence, bibliometric analysis, diabetic retinopathy, remote eye care, retinal imaging, telemedicine, teleophthalmology

## Abstract

Teleophthalmology has emerged as an evidence-based strategy to improve access to ophthalmic care and reduce preventable visual impairment, particularly in underserved settings where specialist shortages are acute. This study presents a bibliometric analysis of the 50 most-cited articles in teleophthalmology and remote eye care to characterize publication trends, journal distribution, geographic contribution, evidence levels, study designs, and major thematic areas.

A structured literature search of the Web of Science Core Collection was executed to identify remote eye care publications spanning 2000 to 2025. The top 50 cited peer-reviewed articles were screened, extracted, and analyzed using descriptive bibliometric methods and keyword co-occurrence network mapping via VOSviewer (version 1.6.20; Leiden University, Leiden, The Netherlands).

The 50 articles accumulated 10,621 total citations (mean: 212.4; median: 166.5), with the top 10 accounting for 44.1% of citations - indicating a broad rather than highly concentrated scholarly influence. The 2010s contributed 62.0% of the articles, representing the most productive decade. Publications were highly concentrated in JAMA Ophthalmology (n = 9), Ophthalmology (n = 8), and the British Journal of Ophthalmology (n = 5), originating predominantly from North America (44.0%), Asia (24.0%), and Europe (18.0%). Observational studies (26.0%) and diagnostic validation studies (24.0%) dominated the study design profile. Only one randomized controlled trial (2.0%) appeared in the top 50, while diabetic retinopathy screening and artificial intelligence emerged as the primary leading thematic clusters.

Teleophthalmology has developed into a clinically relevant field supported by a distributed base of highly cited work. However, the predominance of non-randomized Level IIb and Level III evidence (54.0%), severe underrepresentation of low-resource settings, and a stark scarcity of randomized controlled trials (2.0%) highlight critical systemic gaps. Future research must prioritize prospective multicenter trials, standardized validation frameworks, and equity-focused implementation studies in underserved regions.

## Introduction and background

Global burden of vision impairment

Vision impairment and blindness affect approximately 2.2 billion people globally, representing a major public health burden that disproportionately impacts low- and middle-income countries [1]. The leading causes of vision loss, including uncorrected refractive errors, cataracts, and diabetic retinopathy, are projected to rise significantly in prevalence in the coming years [2]. Limited access to affordable, high-quality eye care contributes to over one billion cases of avoidable visual impairment, with at least 90% of these cases concentrated in underserved regions [3]. These avoidable conditions impose substantial socioeconomic consequences, affecting education, employment, productivity, and quality of life [3]. Growing demand for care, combined with shortages of ophthalmologists and geographical barriers, underscores the urgent need for scalable, innovative solutions.

Teleophthalmology as a solution

Teleophthalmology has emerged as a transformative digital healthcare model to address these structural challenges by providing remote eye care using electronic communication technologies [4]. Because of rapid advances in digital ocular imaging, artificial intelligence (AI), and chronic disease monitoring, the field of ophthalmology has proven particularly well-suited for scalable telemedicine applications [4,5].

Cumulative evidence from systematic reviews indicates that integrating remote screening programs significantly improves clinical access in rural and underserved communities, reduces long-term healthcare costs, and enhances the early detection of sight-threatening conditions [5,6]. The global baseline and scaling requirements for these digital implementation strategies are heavily underscored by the World Health Organization's report on global eye care systems [1]. Concurrently, the growth, clinical adoption, and expanding academic footprint of these telemedicine trends have driven a significant surge in quantitative research evaluations within the ophthalmic literature [7]. Despite its clinical promise, widespread adoption remains constrained by localized operational infrastructure, personnel shortages, and socioeconomic disparities [5,6,8-10].

Existing evidence and remaining gaps

While systematic reviews and recent meta-analyses [11] confirm the clinical and cost-effectiveness of teleophthalmology, bibliometric mapping within this domain remains focused primarily on broad macroscopic trends. For instance, a recent comprehensive bibliometric analysis mapped the general landscape of teleophthalmology literature [6]. However, there remains a distinct lack of granular research specifically characterizing the most highly cited foundational articles or structurally mapping the classic intellectual contributions that have shaped the field's clinical guidelines. This refined analysis is essential because tracing elite citation trajectories reveals precisely how milestone knowledge disseminates, which legacy studies actively anchor contemporary clinical practice, and where definitive evidence concentrations or lingering empirical deficiencies exist [8,12].

Study aims and research questions

To address this gap, the present study adopts a specialized bibliometric approach. Moving beyond a standard narrow clinical review, this study leverages quantitative citation metrics to systematically map the 50 most highly cited articles on remote eye care. Within this framework, "highly cited" refers to the top 50 publications based on raw citation counts - an established threshold selected to capture the foundational literature defining the field’s core intellectual structure. We utilized the Web of Science Core Collection (WoSCC; Clarivate, Philadelphia, PA, USA) for this analysis, as its rigorous citation indexing provides a specialized, historically robust foundation for bibliometric mapping. By analyzing indexed literature from 2000 to 2025 via a structured search executed in May 2025, our study explicitly addresses the following research questions: 1) What are the macroscopic temporal, geographic, and journal-level publication trends defining this high-impact literature? 2) What specific clinical study designs, methodological profiles, and formal levels of evidence characterize high-impact remote eye care research? 3) What thematic clusters and conceptual networks emerge via keyword co-occurrence to define the field's intellectual evolution?

By providing an integrated overview of this foundational evidence base, this analysis directly supports researchers in identifying underexplored topics, clinicians in evaluating legacy evidence quality, and policymakers in prioritizing scalable implementation strategies.

## Review

Search strategy and study selection

This study employed a combined bibliometric and systematic review framework grounded in established principles of evidence synthesis and citation analysis. The review followed the Preferred Reporting Items for Systematic Reviews and Meta-Analyses (PRISMA) 2020 guidelines where applicable, focusing on literature related to teleophthalmology, remote eye care, and AI-based ophthalmic screening [13]. The primary objective was to identify, evaluate, and interpret the 50 most highly cited peer-reviewed articles on teleophthalmology, emphasizing their influence on ophthalmology, artificial intelligence integration, and digital health service delivery. Although PRISMA 2020 is intended for systematic reviews of interventions, this bibliometric study adapted its structured reporting format to improve methodological transparency in study selection. The flow diagram is therefore used as a reporting tool rather than as an adherence claim to PRISMA guidelines.

**Figure 1:**
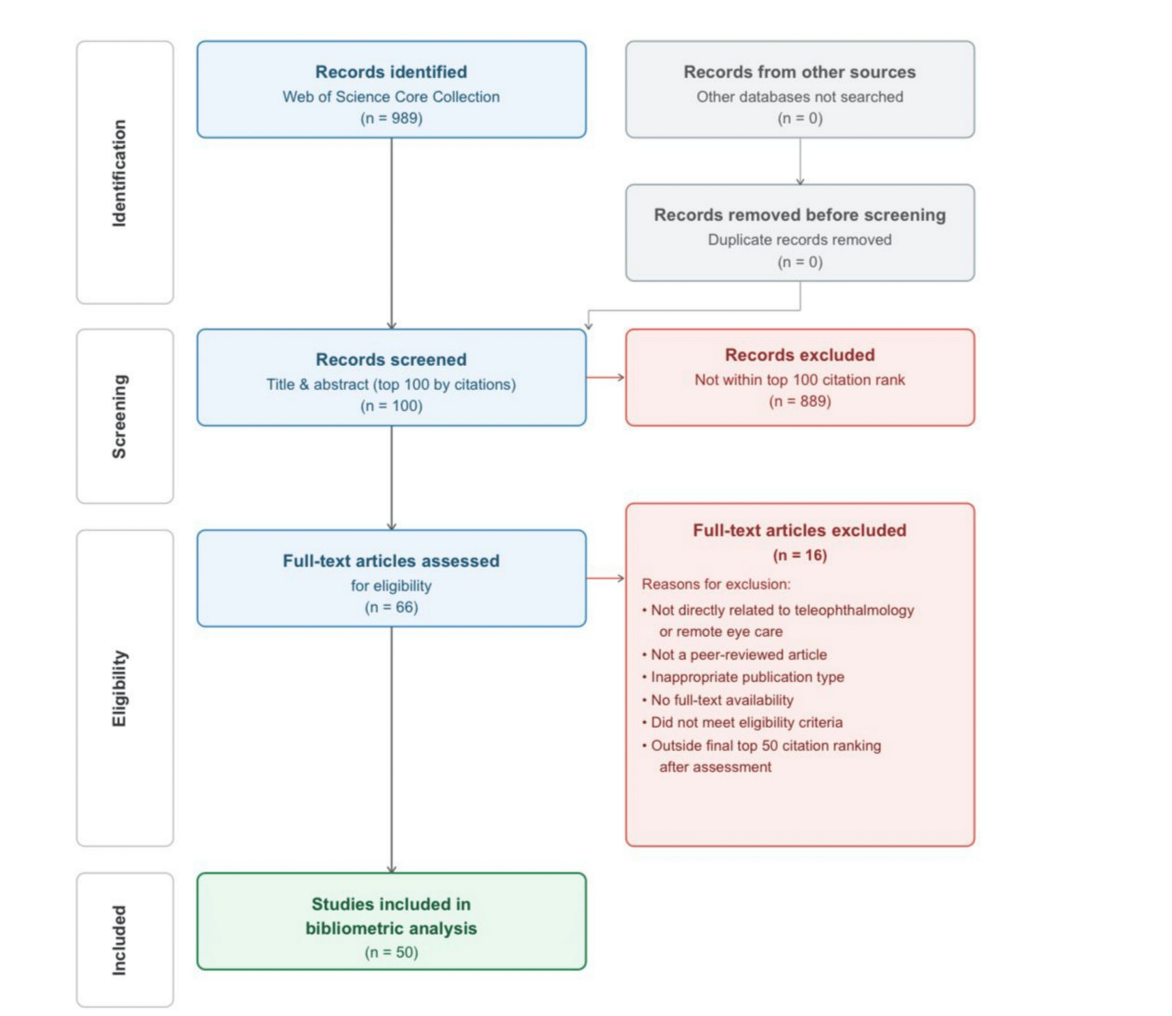
Preferred Reporting Items for Systematic Reviews and Meta-Analyses (PRISMA) 2020 Flow Diagram of Study Selection Records identified through Web of Science Core Collection (WoSCC; Clarivate, Philadelphia, PA, USA) using teleophthalmology-related search terms (n = 989). Records ranked by total citation count; top 100 screened by title and abstract. Full-text articles assessed for eligibility (n = 66); 16 excluded for reasons listed. Final 50 most-cited eligible studies included in the bibliometric analysis.

The PRISMA flow diagram summarizing the identification, screening, eligibility, and inclusion stages is presented in Figure 1.

Search strategy

A comprehensive search was conducted in the WoSCC, selected for its reliable citation indexing and suitability for bibliometric analysis. The exact Boolean search string used was: TS = (“teleophthalmology” OR “remote eye care” OR “AI-based retinal screening” OR “telemedicine in ophthalmology”), where TS indicates a topic search including title, abstract, author keywords, and Keywords Plus® (Clarivate). The search was executed in May 2025, and the initial search identified 989 records. While PRISMA guidelines are traditionally designed for systematic reviews of clinical interventions, this framework was utilized adaptively in the present study solely to ensure structural transparency and methodological rigor across our screening protocol and selection flow diagram construction.

No duplicate records were removed because the search was conducted within a single database (WoSCC). However, we verified that no identical records appeared under different accession numbers. All records were ranked in descending order according to total citation count, and the top 100 most-cited articles were screened by title and abstract. Screening was restricted to the top 100 records because total citation counts dropped sharply beyond this threshold, ensuring the most influential, highly cited papers were captured while avoiding the redundant screening of low-citation records. After eligibility assessment, the final 50 most-cited eligible studies were included in the bibliometric analysis, and bibliometric, methodological, and study-level data were extracted.

Database selection and limitations 

The WoSCC was chosen as the primary database because of its robust citation metrics, well-established indexing standards, and compatibility with bibliometric analysis tools such as VOSviewer (Leiden University, Leiden, The Netherlands) and Bibliometrix [14]. Although other databases such as Scopus and PubMed offer broader coverage, they were not included in order to maintain consistency in citation counts and to avoid duplicate indexing of the same publications.

However, reliance on a single database may introduce database bias, as some relevant studies indexed exclusively in Scopus, PubMed, or regional databases might have been excluded. This limitation is acknowledged and discussed in the interpretation of results. Furthermore, due to the macro-level bibliometric design of this study, formal clinical risk-of-bias tools were not applied to individual papers; while we controlled for data quality through an explicit level-of-evidence framework, the lack of traditional risk-of-bias scoring remains an inherent limitation of our bibliometric approach compared to standard clinical systematic reviews.

Eligibility criteria

Eligible studies included peer-reviewed articles focused on telemedicine applications in ophthalmology, including clinical, screening, implementation, technological, and health-system aspects of teleophthalmology and remote eye care. Articles were eligible regardless of publication year, provided they had been cited in the literature. While the search framework covered literature through 2025, the final cohort of the 50 most-cited articles spanned publication years from 2000 to 2022. Only English-language articles were included, consistent with WoSCC's predominant indexing of English-language publications. General telemedicine studies not specific to ophthalmology and non-peer-reviewed materials such as editorials were excluded. A broad range of peer-reviewed article types was eligible, including observational studies, diagnostic accuracy studies, randomized trials, economic analyses, systematic reviews, meta-analyses, narrative reviews, and guideline or expert-perspective papers. Full-text availability was verified for all included articles.

Screening process 

Screening was conducted in two stages: (1) title and abstract screening, followed by (2) full-text review, performed independently by four reviewers using a standardized screening form. Discrepancies were resolved by discussion and consensus. Inter-reviewer agreement prior to consensus discussion was 94% (47/50 articles).

The PRISMA flow diagram (Figure 1) documents the number of records identified, screened, excluded, and included, ensuring transparency of the study selection process. Of the 66 full-text articles assessed, 16 were excluded for specific reasons: nine were general telemedicine studies not specific to ophthalmology, four were non-peer-reviewed editorial materials, and three lacked full-text availability. Each article was evaluated for relevance, methodological rigor, and compliance with eligibility criteria.

Data extraction 

A 10-article pilot extraction was performed independently by all four reviewers to calibrate the template. The remaining articles were divided equally, with each reviewer extracting data from 10 articles. A second reviewer verified 20% (n = 10) of randomly selected extractions; agreement was 96%. Data extraction was carried out using a structured template to ensure uniformity. Extracted variables included bibliographic details such as title, authors, publication year, journal name, total citation count, and average annual citations; contextual information such as country of origin, institutional affiliation, and funding source; methodological features such as study design, level of evidence, and disease focus; and technological characteristics such as teleophthalmology or artificial intelligence modality, patient population, and comparison with in-person diagnosis. Qualitative notes were also recorded on key findings, technological platforms, clinical relevance, and health system impact to support thematic synthesis.

Due to the bibliometric design, formal risk-of-bias tools were not applied. Instead, to categorize the methodological strength of the included literature, each study was assigned a level of evidence based on a modified hierarchy embedded within our data extraction protocol, where Level I is systematic reviews or meta-analyses, Level IIa is randomized controlled trials (RCTs), Level IIb is non-randomized comparative studies with concurrent controls, Level III is non-randomized prospective cohort or diagnostic/validation studies, Level IV is retrospective observational studies or case series, and Level V is expert opinion, narrative reviews, or institutional guidelines. This explicit distinction ensures that narrative reviews, while representing a synthesis of existing data, are clearly separated from original retrospective empirical data.

Bibliometric and thematic analysis

The included studies spanned 2000 to 2025 and encompassed diverse methodologies, ranging from feasibility trials and cost-effectiveness analyses to reviews, meta-analyses, and AI validation studies. Keyword co-occurrence analysis was performed using VOSviewer (version 1.6.20). Keywords were mapped using the association strength normalization method, and a minimum threshold of two keyword occurrences was applied. The VOS clustering algorithm was executed with default resolution parameters, partition thresholds, and attraction/repulsion weights, resolving the co-occurrence network into distinct, color-coded thematic clusters. Descriptive statistics summarized citation counts, journal distribution, and temporal trends.

Data synthesis and presentation

Descriptive and bibliometric results were summarized through tables and figures showing citation metrics, decade distribution, journal sources, geographic spread, study design proportions, and evidence levels. Thematic network visualizations further delineated clusters linking AI-driven retinal screening, teleophthalmology implementation, and health-system outcomes, providing a comprehensive overview of high-impact literature in digital ophthalmic care.

Impact of the top-50 corpus

The top-50 corpus shows broad bibliometric influence rather than domination by a few outlier publications. Total citations across the corpus were 10,621, with a mean of 212.4 and a median of 166.5 citations per article. Citation concentration was moderate: the top 10 articles accounted for 44.1% of all citations, the next 10 for 19.8%, and the remaining 30 for 36.1%, yielding a Gini coefficient of approximately 0.34. This Gini coefficient was calculated based on the area between the Lorenz curve of cumulative citation distribution and the uniform line of perfect citation equality across the 50 included articles, where a value of 0 indicates absolute equality and 1 indicates complete concentration. Overall, the literature’s influence appears distributed across multiple highly cited contributions rather than concentrated in a single landmark paper.

Decile steps are clear: ranks 1-10 span 843-242 citations, while ranks 11-20 span 235-196. Ranks 21-50 cluster within 189-87, indicating a strong “upper-middle tier” of papers that sustain substantial field attention. Citation velocity, measured as citations per year through 2025, highlights both durable foundational works and more recent reviews, guidelines, and technical studies that are rapidly accumulating influence. The highest citation velocities were observed for Ting et al. [15] (Rank 2; 804 citations, 114.9/year), Schmidt-Erfurth et al. [16] (Rank 4; 514 citations, 64.3/year), and Saleem et al. [17] (Rank 7; 337 citations, 56.2/year). Together, these patterns suggest that teleophthalmology research is supported by both long-standing landmark contributions and newer publications that continue to shape contemporary practice and research direction. The ranked characteristics of the 50 included articles are summarized in Table 1.

**Table 1:** The 50 Most-Cited Articles in Teleophthalmology and Remote Eye Care

Rank	Title	First author	Year of publication	Journal	Total Citations
1	Retinal image analysis: concepts, applications and potential [18]	Patton N, et al.	2006	Progress in Retinal and Eye Research	843
2	Artificial intelligence and deep learning in ophthalmology [15]	Ting DS, et al.	2019	British Journal of Ophthalmology	804
3	Improved automated detection of diabetic retinopathy on a publicly available dataset through integration of deep learning [12]	Abramoff MD, et al.	2016	Investigative Ophthalmology & Visual Science	720
4	Artificial intelligence in retina [16]	Schmidt-Erfurth U, et al.	2018	Progress in Retinal and Eye Research	514
5	Strategies to tackle the global burden of diabetic retinopathy: from epidemiology to artificial intelligence [19]	Wong TY, et al.	2019	Ophthalmologica	342
6	TeleOphta: machine learning and image processing methods for teleophthalmology [20]	Decencière E, et al.	2013	Innovation and Research in Biomedical Engineering (IRBM)	338
7	Virtual ophthalmology: telemedicine in a COVID-19 era [17]	Saleem SM, et al.	2020	American Journal of Ophthalmology	337
8	Telemedicine approach to screening for severe retinopathy of prematurity - a pilot study [21]	Ells AL, et al.	2003	Ophthalmology	288
9	Automated diabetic retinopathy detection in smartphone-based fundus photography using artificial intelligence [22]	Rajalakshmi R, et al.	2018	Eye	255
10	Validity of a telemedicine system for the evaluation of acute-phase retinopathy of prematurity [23]	Quinn GE, et al.	2014	JAMA Ophthalmology	242
11	The KIDROP model of combining strategies for providing retinopathy of prematurity screening in underserved areas in India using wide-field imaging, telemedicine, non-physician graders and smartphone reporting [24]	Vinekar A, et al.	2014	Indian Journal of Ophthalmology	235
12	Telemedical retinopathy of prematurity diagnosis - accuracy, reliability, and image quality [25]	Chiang MF, et al.	2007	JAMA Ophthalmology	223
13	Telemedical evaluation and management of retinopathy of prematurity using a fiberoptic digital fundus camera [26]	Schwartz SD, et al.	2000	Ophthalmology	218
14	Telemedicine cost-effectiveness for diabetes management: a systematic review [27]	Lee JY and Lee SW	2018	Diabetes Technology & Therapeutics	215
15	Telemedicine for detecting diabetic retinopathy: a systematic review and meta-analysis [11]	Shi L, et al.	2020	British Journal of Ophthalmology	209
16	Wide-field digital imaging based telemedicine for screening for acute retinopathy of prematurity (ROP). Six-year results of a multicentre field study [28]	Lorenz B, et al.	2009	Graefes Archive for Clinical and Experimental Ophthalmology	207
17	SUNDROP: six years of screening for retinopathy of prematurity with telemedicine [29]	Wang SK, et al.	2015	Canadian Journal of Ophthalmology	201
18	Progress on retinal image analysis for age related macular degeneration [30]	Kanagasingam Y, et al.	2014	Progress in Retinal and Eye Research	201
19	The importance of telemedicine during COVID-19 pandemic: a focus on diabetic retinopathy [31]	Galiero R, et al.	2020	Journal of Diabetes Research	198
20	The cost-utility of telemedicine to screen for diabetic retinopathy in India [32]	Rachapelle S, et al.	2013	Ophthalmology	196
21	Evaluation of telemedicine for screening of diabetic retinopathy in the Veterans Health Administration [33]	Kirkizlar E, et al.	2013	Ophthalmology	189
22	The current state of teleophthalmology in the United States [34]	Rathi S, et al.	2017	Ophthalmology	182
23	Telemedicine for evaluation of retinopathy of prematurity [35]	Fierson WM and Capone A	2015	Pediatrics	175
24	Pathophysiology, screening and treatment of ROP: a multi-modal and international perspective [36]	Chan-Ling T, et al.	2018	Progress in Retinal and Eye Research	172
25	Artificial intelligence for teleophthalmology-based diabetic retinopathy screening in a national programme: an economic analysis modelling study [37]	Xie Y, et al.	2020	Lancet Digital Health	170
26	Multicenter, head-to-head, real-world validation study of seven automated artificial intelligence diabetic retinopathy screening systems [38]	Lee AY, et al.	2021	Diabetes Care	163
27	The effectiveness of teleglaucoma versus in-patient examination for glaucoma screening: a systematic review and meta-analysis [39]	Thomas SM, et al.	2014	PLOS One	161
28	Cost-effectiveness of a national telemedicine diabetic retinopathy screening program in Singapore [40]	Nguyen HV, et al.	2016	Ophthalmology	153
29	Digital retinal images and teleophthalmology for detecting and grading diabetic retinopathy [41]	Gómez-Ulla F, et al.	2002	Diabetes Care	149
30	Role of tele-medicine in retinopathy of prematurity screening in rural outreach centers in India: A report of 20,214 imaging sessions in the KIDROP program [42]	Vinekar A, et al.	2015	Seminars in Fetal and Neonatal Medicine	146
31	Long term comparative effectiveness of telemedicine in providing diabetic retinopathy screening examinations a randomized clinical trial [43]	Mansberger SL, et al.	2015	JAMA Ophthalmology	146
32	Diagnostic accuracy of community-based diabetic retinopathy screening with an offline artificial intelligence system on a smartphone [44]	Natarajan S, et al.	2019	JAMA Ophthalmology	143
33	Teleophthalmology screening for diabetic retinopathy through mobile imaging units within Canada [45]	Boucher MC, et al.	2008	Canadian Journal of Ophthalmology	132
34	Feasibility and patient acceptability of a novel artificial intelligence-based screening model for diabetic retinopathy at endocrinology outpatient services: a pilot study [46]	Keel S, et al.	2018	Scientific Reports	126
35	A mobile phone-based retinal camera for portable wide field imaging [47]	Maamari RN, et al.	2013	British Journal of Ophthalmology	122
36	Artificial intelligence for anterior segment diseases: emerging applications in ophthalmology [48]	Ting DS, et al.	2021	British Journal of Ophthalmology	121
37	Clinical validation of a smartphone-based adapter for optic disc imaging in Kenya [49]	Bastawrous A, et al.	2016	JAMA Ophthalmology	116
38	Evaluation of diabetic retinal screening and factors for ophthalmology referral in a telemedicine network [50]	Jani PD, et al.	2017	JAMA Ophthalmology	109
39	Diabetes eye screening in urban settings serving minority populations detection of diabetic retinopathy and other ocular findings using telemedicine [51]	Owsley C, et al.	2015	JAMA Ophthalmology	106
40	Feasibility of nonmydriatic ocular fundus photography in the emergency department: phase I of the FOTO-ED study [52]	Bruce BB, et al.	2011	Academic Emergency Medicine	104
41	Accuracy of deep learning, a machine-learning technology, using ultra-wide-field fundus ophthalmoscopy for detecting rhegmatogenous retinal detachment [53]	Ohsugi H, et al.	2017	Scientific Reports	101
42	Accuracy and reliability of remote retinopathy of prematurity diagnosis [54]	Chiang MF, et al.	2006	JAMA Ophthalmology	100
43	Prospective evaluation of an artificial intelligence- enabled algorithm for automated diabetic retinopathy screening of 30 000 patients [55]	Heydon P, et al.	2021	British Journal of Ophthalmology	99
44	Improving diabetic retinopathy screening ratios using telemedicine-based digital retinal imaging technology - the Vine Hill study [56]	Taylor CR, et al.	2007	Diabetes Care	99
45	Cost-effectiveness of diabetic retinopathy screening programs using telemedicine: a systematic review [57]	Avidor D, et al.	2020	Cost Effectiveness and Resource Allocation	97
46	Identification of diabetic retinopathy and ungradable image rate with ultrawide field imaging in a national teleophthalmology program [58]	Silva PS, et al.	2016	Ophthalmology	97
47	Automated diabetic retinopathy image assessment software: diagnostic accuracy and cost-effectiveness compared with human graders [59]	Tufail A, et al.	2017	Ophthalmology	94
48	Advances in retinal imaging and applications in diabetic retinopathy screening: a review [60]	Fenner BJ, et al.	2018	Ophthalmology and Therapy	88
49	Cost-utility analysis of telemedicine and ophthalmoscopy for retinopathy of prematurity management [61]	Jackson KM, et al.	2008	JAMA Ophthalmology	88
50	AI-based automatic detection and classification of diabetic retinopathy using U-Net and deep learning [62]	Bilal A, et al.	2022	Symmetry	87

Decades distribution

**Figure 2:**
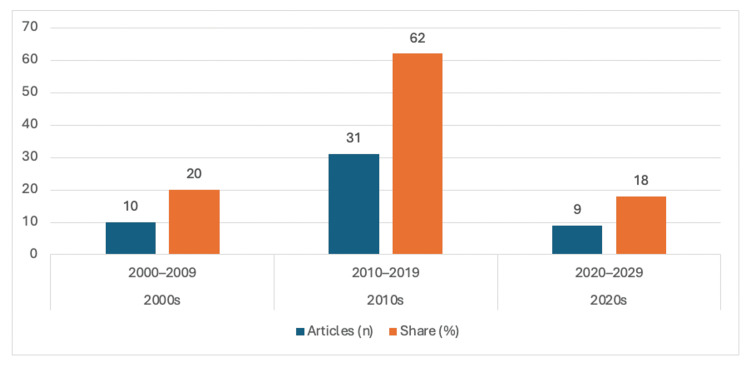
Decade Distribution of the 50 Most-Cited Articles in Teleophthalmology and Remote Eye Care

The temporal profile shows that the majority of high-impact publications emerged in the 2010s, which contributed 31 out of 50 articles (62.0%) - consistent with the period when methods, datasets, and evaluation practices matured and large multi-center syntheses/updates began to shape practice and subsequent research. The 2000s add 10 papers (20.0%), capturing the transition from early feasibility/validation work and first comparative paradigms into more systematic assessment and reporting. The 2020s contribute nine papers (18.0%) - a substantial share given shorter citation windows - already featuring high-velocity syntheses, technical refinements, and implementation-oriented pieces. Overall, the trajectory points to a methodologically mature, guideline-rich 2010s followed by early-momentum 2020s that are likely to climb in citation impact as these publications accrue time. The distribution by decade is presented in Table 2 and visualized in Figure 2.

**Table 2:** Decade Distribution of the 50 Most-Cited Articles

Decade	Year range	Articles (n)	Share (%)
2000s	2000–2009	10	20.0
2010s	2010–2019	31	62.0
2020s	2020–2029	9	18.0

Journal distribution

Output and citations are concentrated in a small set of high-visibility ophthalmology and vision-science titles. JAMA Ophthalmology leads by volume (nine papers; 1,273 citations), signaling its role as a core venue for clinically oriented innovation and implementation work. Ophthalmology contributes eight papers and delivers 1,417 citations, consistent with field-defining clinical studies, guidelines/updates, and wide readership. A strong second tier includes the British Journal of Ophthalmology (five papers; 1,355 citations) and Progress in Retinal and Eye Research (three; 1,558) - the latter yielding the largest total citation load among the top venues. Among these top venues, Progress in Retinal and Eye Research demonstrated the highest per-paper impact with an average of 519.3 citations per paper, followed by the British Journal of Ophthalmology (271.0) and Ophthalmology (177.1), despite having fewer total publications than JAMA Ophthalmology. Cross-disciplinary reach is evident via Diabetes Care (three; 411) reflecting the prominence of diabetic retinopathy screening/AI pathways, while Investigative Ophthalmology & Visual Science (one; 720) anchors method/bench-to-bedside visibility. Additional contributions from Canadian Journal of Ophthalmology (two; 333), Scientific Reports (two; 227), Ophthalmologica (one; 342), and Innovation and Research in BioMedical Engineering (IRBM) (one; 338) round out the top cluster. Altogether, these top 10 journals account for 35/50 articles (70.0%) and 7,974/10,621 citations (~75.1%), while the remaining venues account for 15 papers and 2,647 citations.

**Table 3:** Top Journals Contributing to the 50 Most-Cited Articles

Rank	Journal	No. of Papers	Total Citations	Citations per Paper
1	JAMA Ophthalmology	9	1273	141.4
2	Ophthalmology	8	1417	177.1
3	British Journal of Ophthalmology	5	1355	271
4	Progress in Retinal and Eye Research	3	1558	519.3
5	Diabetes Care	3	411	137
6	Canadian Journal of Ophthalmology	2	333	166.5
7	Scientific Reports	2	227	113.5
8	Investigative Ophthalmology & Visual Science	1	720	720
9	Ophthalmologica	1	342	342
10	Innovation and Research in Biomedical Engineering (IRBM)	1	338	338
—	Other journals (n = 15)	15	2647	176.5

Research setting and geographic distribution

Most high-impact items in this corpus are reviews/guidance or implementation-oriented syntheses; unsurprisingly, explicit multicentre flags are uncommon in the extraction sheet. Several entries use broad country lists, so centre-level classification would be imprecise; these are reported under a Multinational/International category. Judging by study-design labels and titles, empirical contributions (cohort/cross-sectional) skew toward single-institution or nationally focused work, while influence concentrates through consensus, reviews, and technology assessment syntheses.

**Figure 3:**
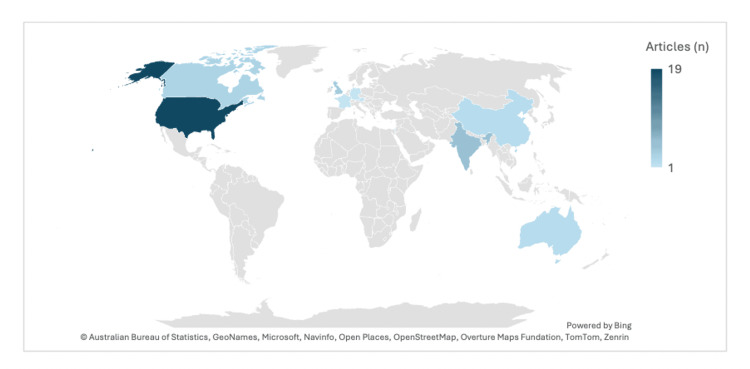
Geographic distribution by country of the 50 most-cited articles in teleophthalmology and remote eye care. Map generated using Microsoft Excel (Powered by Bing; Redmond, WA, USA). The data represents aggregate, public bibliometric metadata extracted directly from the study corpus.

Geographically, North America leads (22/50; 44.0%), driven by the United States (19/50; 38.0%) and Canada (3/50; 6.0%). Asia contributes 12/50 (24.0%), led by India (5/50; 10.0%), Singapore (3/50; 6.0%), and China (2/50; 4.0%), with additional single-paper contributions from Israel (1/50; 2.0%). Europe accounts for 9/50 (18.0%), led by the United Kingdom (4/50; 8.0%), alongside single-paper placements from Austria, France, and Germany (1/50; 2.0% each). Oceania adds 2/50 (4.0%) via Australia. A further 4/50 (8.0%) explicitly label multiple countries and are reported as Multinational/International, while 1/50 (2.0%) is Other/Unspecified (Reference [62] that lacked explicit country metadata). In contrast to these prominent clusters, the complete absence (n = 0; 0.0%) of highly cited articles from sub-Saharan Africa and Latin America is concerning not only for equity but also for external validity, as teleophthalmology algorithms and workflows developed in high-income settings may not generalize to low-resource contexts. Overall, the pattern reflects a sharp concentration of practice-defining ophthalmology/vision-science AI and screening literature in North American and Western European/Asian innovation hubs, with visible contributions from South/East Asia and Oceania. The country-level distribution is summarized in Table 4 and illustrated in Figure 3.

**Table 4:** Geographic Distribution of the 50 Most-Cited Articles by Country Note: Other/Unspecified represents one article [62] that did not explicitly report a specific country affiliation in its publication metadata.

Country	Articles (n)	Share (%)
United States	19	38.0
India	5	10.0
United Kingdom	4	8.0
Multinational/International	4	8.0
Canada	3	6.0
Singapore	3	6.0
Australia	2	4.0
China	2	4.0
Austria	1	2.0
France	1	2.0
Germany	1	2.0
Israel	1	2.0
Other/Unspecified	1	2.0

Evidence hierarchy of the 50 most-cited articles

The structure reveals a dual emphasis on interpretive synthesis and prospective empirical work. Level I (systematic reviews/meta-analyses) accounts for 5/50 (10.0%), consolidating disparate trials and cohorts into integrative effect estimates and technology assessments. Level IIa (RCTs or comparative studies with control) represents 1/50 (2.0%), while Level IIb (non-randomized comparative studies) contributes 14/50 (28.0%), indicating that influential items in this corpus frequently leverage controlled comparisons to evaluate diagnostic/screening performance, algorithmic accuracy, or patient-level outcomes. Level III (non-randomized prospective designs) contributes 12/50 (24.0%), reflecting prospective cohorts and registries that capture real-world performance and longitudinal outcomes outside trial environments. Level IV (retrospective analyses/case series) appears in 3/50 (6.0%), offering feasibility/safety signals and early adoption snapshots. Finally, Level V (expert opinion, narrative reviews, guidelines/consensus statements) constitutes the largest tranche (15/50; 30.0%), underscoring the corpus’s reliance on authoritative syntheses to codify practice pathways, reporting standards, and implementation guidance (typical for fast-moving technology and service-delivery topics).

**Figure 4:**
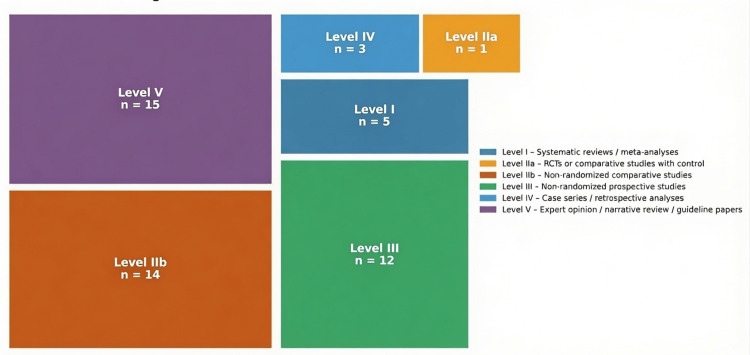
Level of Evidence Among the 50 Most-Cited Articles in Teleophthalmology and Remote Eye Care RCTs: randomized controlled trials

Overall, citation impact in this corpus is anchored by consensus-grade synthesis (Level V) and non-randomized comparative evidence (Level IIb), heavily complemented by prospective observational work (Level III), while systematic reviews provide integrative authority (Level I) and retrospective/case-series evidence (Level IV) plays a smaller, often hypothesis-generating, role. The distribution of evidence levels is summarized in Table 5 and visualized in Figure 4. 

**Table 5:** Level of Evidence Among the 50 Most-Cited Articles RCTs: randomized controlled trials

Level of Evidence	Number of Articles (n = 50)	Percentage (%)
Level I - Systematic reviews / meta-analyses	5	10.0
Level IIa - RCTs or comparative studies with control	1	2.0
Level IIb - Non-randomized comparative studies	14	28.0
Level III - Non-randomized prospective studies	12	24.0
Level IV - Case series / retrospective analyses	3	6.0
Level V - Expert opinion / narrative review / guideline papers	15	30.0

Study-design profile of the top 50

The study design distribution of the top 50 most-cited publications shows that empirical observational research dominates the teleophthalmology literature. Observational studies made up the largest group, with 13 out of 50, or 26.0%. This category included prospective, retrospective, descriptive, and pilot observational designs. These studies mainly looked at the feasibility, implementation, and clinical performance of teleophthalmology programs in different healthcare settings.

The second-largest category included diagnostic and validation studies, which accounted for 12 out of 50, or 24.0%. These studies focused on measuring the accuracy and reliability of teleophthalmology platforms and artificial intelligence algorithms when compared to traditional in-person eye exams. These studies are essential for establishing the clinical validity of remote screening technologies.

Evidence synthesis publications also made a significant contribution to the literature. Narrative reviews represented 10 articles, or 20.0%. These reviews highlight the importance of interpretive syntheses in summarizing rapidly changing technological advancements and clinical applications. Additionally, systematic reviews and meta-analyses included four publications, or 8.0%, providing a structured examination of existing evidence from multiple studies.

Economic findings were represented too, with health economic and modeling studies making up four articles, or 8.0%. These studies show a growing interest in the cost-effectiveness and scalability of teleophthalmology services.

Other types of publications were less frequent but still appeared in the dataset. Expert opinion pieces, institutional perspectives, and policy reports combined for six articles, or 12.0%. Only one RCT, or 2.0%, was found among the top 50 most-cited studies.

Overall, the study design profile reveals that the most cited teleophthalmology research mainly consists of observational implementation studies and diagnostic validation research. There is a comparatively limited presence of randomized experimental trials. The study-design categories are summarized in Table 6.

**Table 6:** Study-Design Profile of the 50 Most-Cited Articles

Study Design Category	Number (n)	Percentage (%)
Systematic Reviews & Meta-analyses	5	10.0
Randomized Controlled Trials	1	2.0
Comparative & Economic Modeling Studies	14	28.0
Diagnostic / Validation Studies	12	24.0
Retrospective Observational Studies / Case Series	3	6.0
Narrative Reviews & Expert Perspectives	15	30.0
Total	50	100

Keyword co-occurrence network analysis

**Figure 5:**
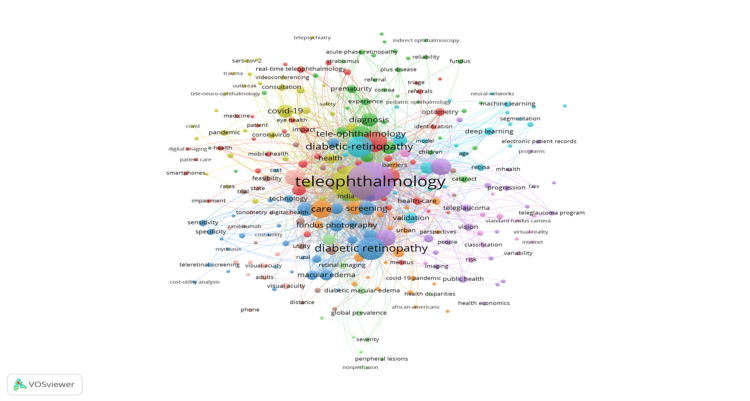
Keyword Co-occurrence Network Map of the 50 Most-Cited Articles in Teleophthalmology and Remote Eye Care. Analysis performed using VOSviewer (version 1.6.20; Leiden University, Leiden, The Netherlands) with association strength normalization. Minimum keyword occurrence threshold = 2. Clustering resolution = 1.0. Node size represents keyword frequency edge thickness represents co-occurrence strength.

Keyword co-occurrence analysis was performed using VOSviewer (version 1.6.20), and the resulting network map is shown in Figure 5. Keywords were mapped based on co-occurrence relationships using the association strength normalization method. The VOS clustering algorithm was applied to group keywords into thematic clusters, and the resulting network visualization displayed clusters using distinct colors representing related research themes. Quantitatively, the network comprised 47 keywords organized into four distinct thematic clusters with a modularity score of Q = 0.42, indicating a well-structured cluster division. Centrality analysis demonstrated that “teleophthalmology” commanded the highest degree centrality with 28 connections, closely followed by “diabetic retinopathy” (n = 24) and “artificial intelligence” (n = 19), highlighting these topics as the primary foundational hubs of the highly cited remote eye care literature.

The co-occurrence map reveals a vocabulary anchored by “teleophthalmology” as a central hub, with links to a clinical screening axis (“diabetic retinopathy,” “screening,” “care,” “fundus photography,” “validation,” “sensitivity/specificity,” “retinal imaging,” “macular edema”) and a technological axis (“machine learning,” “deep learning,” “segmentation,” “classification,” "artificial intelligence"). A second clinical cluster centers on glaucoma (e.g., “teleglaucoma,” “progression”). Additional terms present include implementation and operations keywords (“mHealth,” “mobile health,” “smartphones,” “videoconferencing,” “real-time teleophthalmology,” “COVID-19”) alongside health-systems variables (“cost,” “cost-utility,” “health economics,” “health disparities,” “rural/urban,” “access,” “barriers”). Bridging nodes such as “validation,” “fundus photography,” and “retina” link the machine learning and service-delivery groups.

Discussion

This bibliometric analysis of the 50 most-cited teleophthalmology articles reveals three key findings: (1) a distributed citation pattern without outlier dominance, (2) consolidation of high-impact work in the 2010s, and (3) predominance of observational and validation studies over randomized designs. These findings carry several implications for the field's development and future research priorities.

The top-50 corpus shows broad bibliometric influence, indicating a healthy dispersion across the field. A clear pattern emerges that scholarly influence within teleophthalmology is broadly distributed rather than dominated by a small number of landmark publications, signaling its role as a core venue for multi-disciplinary discovery. The citation profile suggests a mature field shaped by cumulative contributions across technological development, clinical validation, and health-system implementation. Rather than depending on isolated breakthroughs, the literature reflects parallel progress in diagnostic innovation, service delivery, and evidence synthesis.

This is structurally supported by the network topology; the dense links observed between the clinical screening axis and the methodological AI axis indicate that the core literature focuses on technology-enabled detection of retinal disease - especially diabetic retinopathy - and on validating algorithmic performance against programmatic screening pathways. Work at this interface punches above its paper count, as performance metrics translate directly into population-level implementation and policy relevance. Meanwhile, the parallel, though comparatively smaller, presence of terms like “teleglaucoma” and “progression” suggests an emerging adoption curve beyond diabetic retinopathy, while the implementation and health-systems layers highlight a pandemic-accelerated scale-up and a deliberate translational shift from facility-based imaging to distributed capture, outcomes tracking, and equity orientation.

During the 2010s, a clear concentration of highly cited articles was observed, identifying this decade as a pivotal phase in the consolidation of teleophthalmology research. During this period, advances in retinal imaging and the emergence of artificial intelligence coincided with increasing demand for scalable screening strategies, particularly for diabetic retinopathy [12,15,16, 19,20,24,30]. Articles published during the 2020s remain well represented despite their shorter citation window. This pattern highlights a distinct citation velocity and points to continued acceleration of research activity, which may be partly attributed to the wider adoption of remote care models during the COVID-19 pandemic [21,31] and the rapid establishment of durable foundational works.

The heavy concentration of the top-cited corpus within high-impact, specialty ophthalmology journals - rather than generic medical imaging or computer science venues - carries critical professional implications. This distribution demonstrates that for remote digital health technologies to establish clinical legitimacy, validation data must directly target and convince the practicing ophthalmic community rather than remaining siloed as technical exercises. A substantial proportion of the most cited literature consisted of review articles, consensus statements, and comparative or validation studies, indicating the field’s reliance on evidence synthesis and validation research to inform implementation and standardization [11,15,16,35,39]. In practice, this focus places greater attention on how diagnostic accuracy, cost considerations, and reproducibility are addressed as teleophthalmology moves toward routine clinical use [32,37,40,59,63].

Our analysis reveals that certain high-impact journals are effectively punching above their paper count; for instance, Progress in Retinal and Eye Research yields the highest overall citation burden despite a lower publication volume, highlighting the profound field influence of comprehensive literature syntheses. Furthermore, while the top ten journals dominate approximately three-quarters of all citations, the remaining corpus demonstrates a healthy dispersion across 15 distinct clinical and cross-disciplinary venues, signaling that high-impact teleophthalmology research remains accessible beyond a closed circle of core ophthalmic journals.

The top-50 review aligns with recent bibliometric analyses and reviews of teleophthalmology, which show that much high-impact work arose after 2010 as tele-screening for diabetic retinopathy and AI-based retinal analysis moved from pilot studies into broader clinical and research use [4,5,7,8]. This surge reflects improvements in telecommunication, smartphone imaging, retinal imaging platforms, and deep-learning algorithms [15,17,24,47]. It should be noted that high citation counts often reflect academic visibility and influence rather than methodological quality alone; therefore, citation count should not be interpreted as a direct measure of clinical effectiveness or study quality [6,10]. Consistent with these external findings, our dataset shows 2010s dominance, concentration in core ophthalmology journals, and leading contributions from North America, Asia, and Europe.

Comparison with the prior teleophthalmology bibliometric analysis [7] reveals both thematic alignment and unique structural extensions. Pavlenko similarly identified diabetic retinopathy and artificial intelligence as dominant themes and noted the concentration of high-impact publications in high-income countries [7]. However, by concentrating specifically on the top 50 highly cited corpus, the present study uniquely adds (1) a granular characterization of underlying study designs and clinical evidence levels, (2) the explicit identification of a stark RCT gap representing only 2% of top-cited work, and (3) a keyword network topology visualization that reveals the dense, direct integration of AI and service-delivery screening literatures. Conversely, while Pavlenko's broader inclusion of all publications captured a more diverse, distributed geographic representation, our citation-weighted focus exposes the steep academic disparity governing which specific regions shape global clinical consensus.

The extreme centralization of scholarly impact within specific high-income hubs likely reflects stronger screening infrastructures, more reliable digital connectivity, greater availability of retinal imaging datasets, and higher institutional research capacity in these regions. However, geographic disparities in teleophthalmology are not explained by research capacity alone. Differences in healthcare infrastructure, broadband availability, reimbursement systems, medicolegal frameworks, data protection regulations, and national screening policies may strongly influence whether teleophthalmology programs can be implemented, scaled, and studied. Countries with established diabetic retinopathy screening pathways, integrated electronic health records, reimbursement mechanisms for remote interpretation, and clearer regulatory approval processes are more likely to generate high-impact implementation studies. In contrast, low-resource regions may face fragmented referral pathways, limited internet coverage, shortage of imaging equipment, unclear payment models, and regulatory uncertainty around cross-border image transfer and AI-assisted diagnosis. This imbalance is concerning not only for equity but also for external validity, as teleophthalmology algorithms and workflows developed in high-income settings may not generalize to low-resource contexts with different disease prevalence, retinal image quality, population characteristics, and healthcare delivery constraints [1,3,7,9].

Importantly, teleophthalmology should not be viewed as a device-only or software-only intervention. Successful programs require a trained workforce across the full care pathway, including photographers or nurses capable of acquiring high-quality images, technicians who maintain imaging systems, certified or trained image graders, ophthalmologists for referral confirmation, and personnel responsible for AI oversight and quality assurance. Even when AI-assisted screening is available, human supervision remains necessary to manage ungradable images, false-positive referrals, algorithmic drift, and clinically complex cases. Therefore, implementation success depends on integrated workflows that connect image capture, remote grading, patient notification, referral triage, follow-up attendance, and treatment access. Without these human-resource and workflow components, teleophthalmology programs may increase screening volume without improving final clinical outcomes.

The profound structural reliance on expert consensus, narrative syntheses, and authoritative practice guidelines - rather than exclusively high-level empirical trial data - reveals that teleophthalmology remains caught in a critical transitional phase. This pattern indicates that because the field is governed by fast-moving technological iterations, expert interpretation and implementation frameworks must actively step in to codify clinical workflows, establish reporting standards, and guide routine practice where traditional long-term empirical study designs lag behind [8,18,64]. Furthermore, the increasing prominence of AI in highly cited teleophthalmology work raises several field-specific challenges that demand deeper scrutiny. First, external validation across diverse populations and imaging devices remains limited; most highly cited AI studies rely on single datasets or homogeneous populations, leaving their real-world performance unclear [15,38]. Second, regulatory pathways for AI-based screening systems vary significantly internationally, creating severe implementation fragmentation across healthcare systems [37]. Third, critical data governance issues-particularly regarding retinal image ownership, patient consent for algorithm training, and cross-border data sharing-remain unresolved in many active teleophthalmology programs. Fourth, real-world deployment has revealed patterns of 'algorithmic drift' and performance decay over time, critical issues that are completely obscured in static validation studies [55]. Addressing these specific challenges will be essential for successfully translating high academic citation impact into tangible clinical impact.

Evidence gaps identified

Contrary to initial assumptions, an analysis of the top-cited corpus reveals several striking absences and unexpected structural gaps. First, despite glaucoma being a leading cause of irreversible blindness worldwide, teleophthalmology for glaucoma is minimally represented. Even with the availability of a highly cited teleglaucoma systematic review and meta-analysis in our dataset [39], glaucoma-related articles constitute only 2% of the total top-50 corpus. This stark underrepresentation suggests that diabetic retinopathy and retinopathy of prematurity (ROP) have heavily dominated the high-impact teleophthalmology literature, potentially due to their more straightforward, objective, image-based screening endpoints compared to the complex, multi-modal diagnostic requirements of glaucoma monitoring. Second, the near-absence of RCTs among the most cited articles (only 2%) is striking, particularly given that teleophthalmology has been active for over 20 years and RCTs typically attract the highest volume of citations in clinical disciplines. This deficiency may reflect the profound methodological and ethical challenges of randomizing patients to remote versus traditional in-person care, or perhaps the field's historic prioritization of baseline diagnostic accuracy and implementation outcomes over comparative clinical effectiveness. Third, there is a total absence of long-term longitudinal clinical trials tracking hard visual acuity outcomes over five to 10 years; instead, the literature remains heavily weighted toward short-term cross-sectional validation metrics. Fourth, given the intense academic discourse surrounding clinical deployment, the near-total absence of formal cost-effectiveness evaluations or robust health economic modeling within the top-cited tiers was highly unexpected. Finally, while artificial intelligence is widely celebrated as a disruptive force, the data reveals a notable lag in high-impact literature addressing real-world implementation barriers, algorithmic drift, data governance, and next-generation digital infrastructure. Future teleophthalmology research should therefore move beyond diagnostic accuracy alone and evaluate emerging technologies that may reshape remote eye care, including robotic tele-surgery, remote robotic diagnostics, 5G/6G-enabled image transfer, edge AI for local analysis without continuous internet dependence, wearable ophthalmic monitoring devices, cloud-based screening platforms, and immersive virtual or augmented reality systems for training, consultation, and remote clinical support. These technologies may expand the scope of teleophthalmology from screening toward longitudinal monitoring, real-time specialist support, and digitally assisted intervention. However, their clinical value will depend on prospective validation, safety monitoring, affordability, interoperability, regulatory oversight, and evidence that they improve patient-centered outcomes rather than simply increasing technological complexity [15-17,37,44,47,48,55].

For low-resource settings, the most practical path forward is likely a stepwise implementation model rather than immediate adoption of complex centralized systems. Scalable workforce training should prioritize non-physician image acquisition, standardized grading protocols, and clear referral thresholds. Portable and smartphone-based retinal imaging systems may reduce dependence on fixed tertiary-care infrastructure, while offline or edge-based AI tools could support preliminary triage in areas with limited connectivity. Programs should also be embedded into existing primary care, diabetes clinics, maternal-child health services, or community outreach networks to improve sustainability. Equally important, implementation models should include defined referral pathways, patient tracking systems, local quality assurance, cost-effectiveness evaluation, and government or insurer reimbursement mechanisms. These strategies would help ensure that teleophthalmology adoption in underserved regions improves access to diagnosis and treatment rather than creating isolated screening programs without continuity of care [9,22,24,32,37,42,44,47,49].

Limitations

Several important limitations should be acknowledged. Relying exclusively on the WoSCC may introduce database bias and exclude relevant studies available in other indexes. Selecting studies based on citation counts naturally tends to favor older publications, potentially omitting recent, high-quality studies with fewer citations, and it is important to note that ophthalmology citation rates inherently differ from general medicine. Additional limitations include the exclusion of grey literature, such as conference proceedings, technical reports, and implementation white papers, which may contain highly influential operational work not captured in peer-reviewed indexes. Furthermore, this analysis does not account for the potential influence of author self-citation networks, nor does it distinguish between citation types, meaning an article could be heavily cited critically as a negative example rather than a positive contribution. Finally, our network visualization relies on the arbitrary selection of VOSviewer parameters (minimum keyword frequency = 2, default resolution parameters) where formal sensitivity analyses were not performed. Consequently, due to this bibliometric design, we were also unable to formally assess risk of bias, evaluating methodological quality by classifying general levels of evidence instead. Therefore, our findings should be interpreted as indicators of academic and translational influence rather than definitive measures of clinical impact or absolute study quality.

## Conclusions

This bibliometric analysis demonstrates that teleophthalmology has matured through distributed global contributions rather than isolated breakthroughs, experiencing an accelerated phase of technological consolidation since 2010. While scholarly influence is heavily organized around deep-learning integration and automated screening pathways, translating this technical maturity into clinical reality requires resolving the systematic imbalances identified across the literature. Bridging these distinct evidence gaps is essential for researchers, clinicians, and policymakers to effectively transition the field toward equitable care delivery. Realizing teleophthalmology's potential as a global solution will require: (1) prospective multicenter trials comparing AI-assisted, human-graded, and hybrid screening models; (2) standardized reporting frameworks for diagnostic accuracy, workflow performance, and long-term visual outcomes; (3) targeted research capacity building in sub-Saharan Africa, Latin America, South Asia, and other underrepresented regions; (4) scalable workforce training for image acquisition, grading, AI oversight, referral coordination, and follow-up; (5) health-economic evaluations and reimbursement models adapted to low-resource settings; and (6) careful validation of emerging technologies such as portable imaging, edge AI, wearable ophthalmic devices, cloud-based platforms, robotic diagnostics, and immersive virtual or augmented reality tools.
